# Narrowing the Genetic Causes of Language Dysfunction in the 1q21.1 Microduplication Syndrome

**DOI:** 10.3389/fped.2018.00163

**Published:** 2018-06-05

**Authors:** Antonio Benítez-Burraco, Montserrat Barcos-Martínez, Isabel Espejo-Portero, Maite Fernández-Urquiza, Raúl Torres-Ruiz, Sandra Rodríguez-Perales, Ma Salud Jiménez-Romero

**Affiliations:** ^1^Department of Spanish, Linguistics, and Theory of Literature, University of Seville, Seville, Spain; ^2^Laboratory of Molecular Genetics, University Hospital “Reina Sofía”, Córdoba, Spain; ^3^Maimónides Institute of Biomedical Research, Córdoba, Spain; ^4^Department of Spanish Philology, University of Oviedo, Oviedo, Spain; ^5^Molecular Cytogenetics Group, Centro Nacional Investigaciones Oncológicas, Madrid, Spain; ^6^Department of Psychology, University of Córdoba, Córdoba, Spain

**Keywords:** chromosome 1q21.1 duplication syndrome, cognitive delay, language deficits, speech problems, CDH1L, ROBO1

## Abstract

The chromosome 1q21.1 duplication syndrome (OMIM# 612475) is characterized by head anomalies, mild facial dysmorphisms, and cognitive problems, including autistic features, mental retardation, developmental delay, and learning disabilities. Speech and language development are sometimes impaired, but no detailed characterization of language problems in this condition has been provided to date. We report in detail on the cognitive and language phenotype of a child who presents with a duplication in 1q21.1 (arr[hg19] 1q21.1q21.2(145,764,455-147,824,207) × 3), and who exhibits cognitive delay and behavioral disturbances. Language is significantly perturbed, being the expressive domain the most impaired area (with significant dysphemic features in absence of pure motor speech deficits), although language comprehension and use (pragmatics) are also affected. Among the genes found duplicated in the child, *CDH1L* is upregulated in the blood of the proband. *ROBO1*, a candidate for dyslexia, is also highly upregulated, whereas, TLE3, a target of FOXP2, is significantly downregulated. These changes might explain language, and particularly speech dysfunction in the proband.

## Introduction

Copy number variant (CNV) syndromes entailing language impairment provide important evidence of how changes in gene dosage impact on the wiring and function of brain areas involved in language processing. The 1q21.1 region contains several segmental-duplication blocks which favor the occurrence of microdeletions and microduplications. The chromosome 1q21.1 duplication syndrome (OMIM# 612475) results from microduplications of the BP3-BP4 region, with a minimum duplicated region of ~1.2 Mb of unique DNA sequence, which includes at least seven genes, although a less common variant, resulting from microduplications of the BP2-BP4 region, with a duplicated region of ~2 Mb and encompassing the region deleted in the TAR syndrome (OMIM# 274000), has been also reported (1). These two variants are commonly referred as Class I 1q21.1 duplication syndrome and Class II 1q21.1 duplication syndrome, respectively.

Many of the subjects bearing the 1q21.1 microduplication exhibit autistic features, attention deficit-hyperactivity disorder, mild or moderate mental retardation, relative macrocephaly, mild dysmorphic features, heart disease, and hypotonia (1–3). Some of these features are shared with carriers of the reciprocal deletion, including cognitive dysfunction, motor problems, articulation deficits, and hypotonia (4). Interestingly, microcephaly is prevalent in deletion carriers, whereas macrocephaly is common in duplication carriers; likewise, deletions are usually associated with schizophrenia, while carriers of the duplication usually exhibit autistic features [(4) and references herein]. Interestingly too, the adult phenotype seems to be milder than the child presentation of the syndrome and includes macrocephaly and abnormalities of possible connective tissue origin, and occasionally, schizophrenia (3). Overall, because the microduplication (as well as the reciprocal deletion) is associated with a highly variable phenotype, and because it exhibits an incomplete penetrance and variable expressivity, it has been suggested that this is a susceptibility locus, rather than a clinically distinct syndrome, which predisposes to neurodevelopmental problems (5).

Little is known about the genetic causes of the neuropsychiatric phenotype of 1q21.1 duplications. The distinctive genomic architecture of the 1q21.1 region, which accounts for its genomic instability, seems to be evolutionarily advantageous, and includes multiple human-specific changes (compared to extant primates) important for brain evolution, such as the expansion of genes coding for proteins with DUF1220 protein motifs, and the duplicative transpositions of non-1q21.1 genes, like *SRGAP2* and *HYDIN* (6). According to Dolcetti et al. (3), gene expression data suggest that candidate genes for the distinctive cognitive profile of people with the 1q21.1 microduplication may include *BCL9, GJA8, PDZK1*, and *PRKAB2*. Nonetheless, no conclusive genotype-phenotype correlations have been found. This is particularly true of the language problems that are commonly observed in patients, among other reasons, because we still lack a comprehensive characterization of the language phenotype associated to this condition.

In this paper, we report on a boy with a microduplication of the 1q21.1 region and provide a detailed description of his language (dis)abilities. Relying on our current knowledge of the genetic aspects of language development and language evolution, but also on *ad-hoc* analyses of the expression pattern of selected genes in the blood of our proband, we propose *BCL9* and *CDH1L* as principal candidates for language dysfunction in this syndrome.

## Material and methods

### Linguistic, cognitive, and behavioral assessment

The global developmental profile of the proband was evaluated with the Spanish versions of the Battelle Developmental Inventories (7), the Luria-Nebraska Neuropsychological Battery for Children (LNNB-C) (8), the Kaufman Assessment Battery for Children (K-ABC) (9), and the Inventory for Client and Agency Planning (ICAP) (10). The Spanish version of the Autism Diagnostic Observation Schedule- Revised (ADOS-R) was used, specifically, for screening autistic features in the child.

#### Battelle developmental inventories

Battelle Developmental Inventories consist of 341 items and are aimed to evaluate personal/social development, adaptive capabilities, motor abilities (gross and fine), communication skills (in the receptive and expressive domains), and cognitive development.

#### Luria-nebraska neuropsychological battery for children (LNNB-C)

The LNNB-C is aimed to evaluate (and anticipate) the child's learning, experience, and cognitive skills, as well as his neuropsychological deficiencies. The test comprises 10 scales that are aimed to measure executive functioning in several areas of interest (manual mobility, left-right orientation, gestures and praxias, verbal regulation, spatial orientation) as well as language functioning in different domains (object and drawing naming, phonological awareness, vocabulary in pictures, similarities and dissimilarities, and numerical operations).

#### Kaufman assessment battery for children (K-ABC)

The K-ABC is aimed to evaluate diverse processing and cognitive abilities as they are put into use, either simultaneously or sequentially, to resolve specific problems. The test comprises 16 subtests arranged into three scales: Simultaneous Processing, Sequential Processing, and Knowledge.

#### Inventory for client and agency planning (ICAP)

The ICAP is aimed to evaluate the subject's functional abilities and maladaptive behaviors in the following general areas: motor skills, social and communication skills, personal living skills, and community living skills. The ICAP measures the frequency and severity of eight types of behavioral disturbances, which are organized in three subscales: asocial maladaptive behavior (uncooperative behavior and socially offensive behavior), internalized maladaptive behavior (withdrawn or inattentive behavior, unusual or repetitive habits, and self-harm), and externalized maladaptive behavior (disruptive behavior, destructive to property, and hurtful to others). Behavior is rated as normal or abnormal, whereas behavioral problems are subsequently rated as marginally serious, moderately serious, serious, or very serious.

#### Autism diagnostic observation schedule-revised (ADOS-R)

This test is designed for toddlers between 18 and 60 months and comprises 23 questions. A positive score is indicative of the possibility of suffering from the disease.

Regarding the language profile of the child, his abilities were evaluated in detail with the Prueba de Lenguaje Oral de Navarra- Revisada [Navarra Oral Language Test, Revised] (PLON-R) (11), and the Spanish version of the Peabody Picture Vocabulary Test, Third Edition [PPVT-3; (12)].

#### Prueba de lenguaje oral de navarra-revisada (PLON-R)

This test was used to assess in depth the child's expressive abilities. The PLON-R is aimed for children between 3 and 6 years, and characterizes language production both structurally and functionally. The composite score “form” evaluates speech sound production at the level of single word, phonological awareness, as well as morphological and syntactic features of the child's imitated and elicited verbal production. The composite score “contents” assesses word and sentence meanings. Finally, the test also evaluates different aspects of language use (planning, autoregulation, and modification under environmental cues) in semi-spontaneous conditions.

#### Peabody picture vocabulary test (PPVT-3)

This test was used to evaluate in more detail the receptive abilities of the proband. The PPVT-3 assesses the correct acquisition of words by the child via several tasks in which the child is asked to point to one of four color pictures on a page after hearing a word.

Additionally, language in use and overall communication skills in spontaneous conditions were assessed through the systematic analysis of a 17-min sample of the child's talk in interaction with his mother. The conversation was video-recorded and then transcribed and coded using CHAT (*Codes for the Human Analysis of Transcripts*) software, a tool of the CHILDES Project (13). CHAT allows to code for speech production phenomena (including information about articulation, prosody, and fluency) in the main lines of the transcript, and for phonological, morphological, syntactic and pragmatic phenomena in dependent lines.

It has to be pointed out that, when evaluating language in use, every linguistic phenomenon is to be assessed from a pragmatic perspective (14). Thus, we first tagged the linguistic errors made by the child indicating the linguistic components affected (phonology, morphology, syntax, lexical semantics). Then we evaluated the communicative effects of each error and labelled it accordingly on the pragmatic level, using the Pragmatic Evaluation Protocol for the analysis of oral Corpora (PREP-CORP), which has been satisfactorily used to provide the linguistic profile of other neurogenic disorders (15–17). The number of occurrences of each labeled phenomenon was automatically computed by means of CLAN (*Computerized Language Analysis*) (18), allowing us to build up a global description of the pragmatic linguistic profile of the proband, that is to say, of the most salient phenomena of his language in use.

Finally, the linguistic profile of the proband's parents was assessed with the verbal scale of the Spanish version of the WAIS-III (19), the Test de Aptitud Verbal “Buenos Aires” [Buenos Aires Verbal Aptitude Test] (BAIRES) (20), the Batería para la Evaluación de los Procesos Lectores en Secundaria y Bachillerato [Battery for the evaluation of reading abilities in high school students] (PROLEC-SE) (21), and one specific task aimed to evaluate the comprehension of passives and correferences.

#### WAIS-III

The verbal scale of the WAIS-III comprises six tasks aimed to assess the subject's abilities in two different domains of language processing: verbal comprehension [Similarities (S), Vocabulary (V), Information (I), and Comprehension (CO)] and working memory [Digits (D) and Arithmetic (A)].

#### BAIRES

The BAIRES is aimed to evaluate language comprehension and production in subjects above 16 years old. It comprises two tasks which assesses the subject's ability to understand and provide with synonymic words and verbal definitions, respectively.

#### PROLEC-SE

The PROLEC-SE is aimed to assess the reading abilities by adolescents between 12 and 18 years old. It comprises six tasks which evaluate three different processes involved in reading: lexical (word reading, pseudoword reading), semantic (text comprehension, text structure), and syntactic (picture-sentence mapping, punctuation marks).

#### *Ad-hoc* task

The *ad-hoc* task was a sentence-picture-matching task aimed to evaluate the comprehension of bound anaphora in complex sentences (that is, the ability to properly bind a determiner phrase in the subordinated clause to a referential element in the main clause) and of canonical long passive structures (that is, with a *by*-phrase). While the former task enquires about highly-demanding computational abilities in the domain of language processing, the latter is usually difficult for people with poor reading abilities, because in Spanish passives are much more frequent in the literary register.

### Molecular and cytogenetic analysis

DNA from the patient and his parents was extracted from 100 μl of EDTA-anticoagulated whole blood using MagNA Pure (Roche Diagnostics, West Sussex, UK) and used for subsequent analyses.

#### Karyotype analysis

Peripheral venous blood lymphocytes were grown following standard protocols and collected after 72 h. A moderate resolution G-banding (550 bands) karyotyping by trypsin (Gibco 1x trypsin® and Leishmann stain) was subsequently performed. Microscopic analysis was performed with a Nikon® eclipse 50i optical microscope and the IKAROS Karyotyping System (MetaSystem® software).

#### Fragile X syndrome analysis

CGG expansions affecting the gene *FMR1* (the main determinant for Fragile X syndrome) were analyzed in the patient according to standard protocols. Polymerase chain reaction (PCR) of the fragile site was performed with specific primers for the fragile region of the *FMR1 locus* and the trinucleotide repeat size of the resulting fragments was evaluated by electrophoresis in agarose gel.

#### Multiplex ligation-dependent probe amplification (MLPA)

MLPA was performed to detect abnormal CNV of the subtelomeric regions of the probands' chromosomes. MLPA is based on the amplification (by use of a single PCR primer pair) of many different probes, each of which detecting a specific subtelomeric DNA sequence. Three different kits from MRC-Holland were used: SALSA® MLPA® probemix P036-E3 SUBTELOMERES MIX 1, SALSA® MLPA® probemix P070-B3 SUBTELOMERES MIX 2B, and SALSA® MLPA® probemix P245-B1 Microdeletion Syndromes-1A. The PCR products were analyzed by capillary electrophoresis in an automatic sequencer Hitachi 3500 and further analyzed with the Coffalyser V 1.0 software from MRC-Holland.

#### Microrrays for CNVs search and chromosome aberrations analysis

The DNA from the patient and his parents was hybridized on a CGH platform (Agilent Technologies). The derivative log ratio spread (DLRS) value was 0.172204. The platform included 60.000 probes. Data were analyzed with Agilent CytoGenomics 3.0.5.1 and qGenViewer, and the ADM-2 algorithm (threshold = 6.0; abs (log2ratio) = 0.25; aberrant regions had more than 4 consecutive probes).

#### qPCR

The expression pattern of genes of interest was assessed in blood by qRT-PCR in order to determine possible differences between the proband and his parents. Total RNA extraction and qRT-PCR were done as previously described (22). Briefly, total RNA was extracted from cells using Trizol (Sigma Aldrich) according to the manufacturer's procedure, followed by a treatment with RNase-free DNase (Roche). cDNA was synthesized from 1 μg of total RNA using a Superscript III First Strand cDNA synthesis kit (LifeTechnologies). Amounts of specific mRNAs in samples were quantified by qRT-PCR using an ABIPrism 7900 HT Detection System (Applied Biosystems) and SYBR green detection. PCR was performed in 96-well microtest plates (Applied Biosystems) with 0.5 units of Taq Polymerase (Applied Biosystems) per well and 35–40 cycles. In all experiments, mRNA amounts were normalized to the total amount of cDNA by using amplification signals for hGUSB. Each sample was determined in triplicate, and at least three independent samples of each patient were analyzed. Primer sequences and PCR conditions are listed in Supplementary Table 1.

Ethics approval for this research was granted by the Comité Ético del Hospital “Reina Sofía.” Written informed consent was obtained from the proband's parents for publication of this case report and of any accompanying tables and images.

## Results

### Clinical history

**Figure 1:**
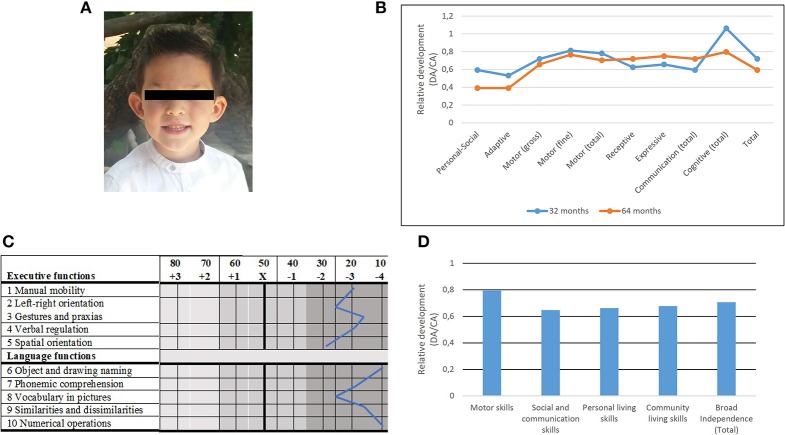
Main physical and cognitive findings in the proband. **(A)** Facial picture illustrating some of the dysmorphological features found in the proband. Written informed consent was obtained from the parents of the child for the publication of this image. **(B)** Developmental profiles of the proband at 2 years and 8 months (blue) and at 5 years and 4 months (orange) according to the Battelle Developmental Inventories. In order to make more reliable comparisons, the resulting scores are shown as relative values referred to the expected scores according to the chronological age of the child. DA, developmental age; CA, chronological age. **(C)** Developmental profile of the proband at age 5 years and 6 months according to Luria-Nebraska Neuropsychological Battery for Children (LNNB-C). DA, developmental age; CA, chronological age. **(D)** Developmental profile of the proband at age 5 years and 6 months according to the Inventory for Client and Agency Planning (ICAP).

The proband (Figure 1A) is a boy born by normal delivery after 37 weeks of risk gestation because of placenta previa with bleeding and threatened abortion. The mother was a 28-year-old woman, who suffered from high blood pressure and diabetes mellitus during pregnancy and who consumed anxiolytic drugs during the first trimester. At the delivery, no signs of disease were observed in the newborn. The child suffered from recurrent infantile colic during the first months of life, as well as from irritability that seriously disturbed his sleeping. At age 2 years and 3 months, his weight was 9.490 kg (percentile 1), his height 86.5 cm (percentile 18) and the occipitofrontal circumference 41.2 cm (below percentile 1). At present, when he is 6 years old, his weight is 19 kg (percentile 21), his height 117 cm (percentile 62) and the occipitofrontal circumference 50 cm (around percentile 30). Cranial magnetic resonance imaging (MRI), performed at 5 years and 7 months of age, yielded normal results. The child was set twice a tympanic drainage in conjunction with adenoidectomy (at 3 years and 4 years). At that time, the Eustachian tube was reported to be underdeveloped, although no auditory impairment was observed. One paternal cousin of the proband is dyslexic, whereas a second paternal cousin (from a different brother of the father) suffers from language developmental delay. Finally, the child exhibits facial dysmorphisms, including mild hypertelorism, small nose, and protruding ears.

### Language and cognitive development

Developmental anomalies were first reported by the teachers of the nursery school when the proband was 2 years old. The child was unable to stand up without aid and fine and gross motor abilities seemed severely delayed. Bladder and bowel control had not been achieved either. The child only babbled and no single word was observed. Stereotypic behavior was reported. The child received early childhood intervention from age 2 years and 4 months. At age 2 years and 8 months his global development was assessed in detail with the Spanish version of the Battelle Developmental Inventories. The resulting scores were suggestive of a mild developmental delay, mostly impacting on his language, social and adaptive abilities, whereas his cognitive skills were slightly above the mean (Figure 1B). When he was 3 years old, the boy started attending a normal nursery school.

At age 5, the parents reported a generalized cognitive and behavioral regression. Stereotypic behaviors increased and the child exhibited lack of cognitive flexibility, occasional spatial disorientation, obsessive behavior, perseverations, unmotivated fears to darkness or load sounds, and lack of interaction with his peers at school. Although he seemed to have mastered a good vocabulary, he failed to put it into use for normal interaction. He also had problems with the conceptualization of time. Moreover, although he received speech and cognitive aid and therapy on a daily basis, at school he failed in achieving the learning objectives planned for that age.

At age 5 years and 4 months, the Spanish version of the Battelle Developmental Inventories was administered again. The obtained scores were not suggestive of a generalized regression, although the child's cognitive skills seemed more impaired than at younger ages (Figure 1B). The Spanish version of the Kaufman Assessment Battery for Children was also administered to evaluate his learning abilities. The most impaired areas were the general mental processing abilities (very significant deficit) and the sequential processing abilities (significant deficit), although the child scored lower than his peers in the remaining domains too. These results reinforce the view that the widespread regression reported by the parents was not for real, and that the child's poor performance in daily tasks, which becomes more evident as the boy grows older, might result from a moderate learning deficit caused by his linguistic and cognitive deficits. Additionally, in view of the behavioral problems reported by parents and teachers, but also of the scores obtained in the Batelle Developmental Inventories, the Spanish version of the Autism Diagnostic Observation Schedule- Revised (ADOS-R) was administered at the age of 5 years and 5 months. The child did not fulfill the requirements for Autism Spectrum Disorders (ASDs). He scored normally in all the tasks, including the use of non-verbal aspects of social interaction, interactive symbolic play, reciprocity during interaction and play, and understanding of, and response to, others' beliefs. The boy usually paid attention to others and was able to express his own feelings toward them and about himself. What is more, the qualitative analysis of the interactive aspects of the spontaneous conversation with his mother confirmed the absence of autism. In truth, the autistic-like behaviors initially reported by parents and teachers (avoidance behaviors, obsessive behaviors, lack of visual contact) were mostly observed when the child was placed in an unfamiliar environment and felt stressed, unsecure, or anxious. It is also possible that he suffered from some sort of compulsive-obsessive disorder.

When the child was 5 years and 6 months old, his global linguistic and cognitive profile, as well as his learning abilities were also evaluated with the Luria-Nebraska Neuropsychological Battery for Children (LNNB-C). The obtained scores were suggestive of a deep impairment of language and executive functions (in all the assessed domains the scores corresponded to percentiles 1–2) (Figure 1C). Likewise, the Inventory for Client and Agency Planning (ICAP) was administered for assessing the child's adaptive behavior. The obtained scores (Figure 1D) were indicative of a delay of nearly 2 years in his global development, being social and communication skills the most impaired domain.

Because of the significant impairment and delay observed in the language domain, we also evaluated in detail the linguistic abilities of the proband at the age of 5 years and 6 months. The global score obtained in the Prueba de Lenguaje Oral de Navarra-Revisada (PLON-R), which evaluates speech sound production at the single word level as well as expressive abilities, was suggestive of a mild delay in the acquisition of the expressive component of language, being phonological awareness and morpho-syntax the most impaired domains. Likewise, a mild delay was suggested regarding the use of language. On the contrary, the child scored like their peers regarding the meaning of words and sentences. Concerning his receptive abilities in the domain of language, the global score obtained in the Spanish version of the Peabody Picture Vocabulary Test (PPVT-3) was suggestive of a delay of 12 months in the acquisition of the meaning of words, with an expected negative impact of his comprehension abilities.

Lastly, we examined in detail the child's language in use and his communication skills through the analysis of a naturally-occurring interaction with his mother. The proband's speech production abilities in natural settings were severely impaired, hindering the child's intelligibility and communication success. Fluency problems included syllabified and effortful articulation, reduplication of syllables in any position of the word, and severe alterations of rhythm, use of pauses, volume, and prosody. Assimilation of specific sounds was frequent in the boy's speech, as well as simplifications of consonant clusters and deletions of weak syllables. Omissions of strong syllables (those bearing the primary stress) and of sounds in initial position were occasionally observed too. Concerning language structure and complexity, the child's mean length of utterance in words (MLUw) was 2.6, which is within the range observed in children aged between 2 years and 6 months, and 2 years and 11 months (23), and thus remarkably lower than the expected value by age. In longer utterances, the child produced morpho-syntactic errors, such as additions or substitutions of prepositions, or omissions of weak pronouns (usually, personal pronouns functioning as the object of the sentence [*me* “me” vs. *yo* “I”]), which may be due to the widespread weak-syllable deletions observed in his discourse. Finally, his interactive pragmatic skills seemed to be preserved: visual contact and joint attention mechanisms were normal, as well as patterns of turn taking and construction of collaborative turns during conversation. However, enunciative pragmatic skills (that is, principles of language use regulating how utterances are interpreted in their contexts of usage), such as the observation of maxims of quality (“be truthful and do not give information that is false or that is not supported by evidence”) and relation (“be relevant, and say things that are pertinent to the discussion”) (24), were slightly impaired, which might be related to his attention problems and/or intellectual disability.

### Cytogenetic and molecular analyses

**Figure 2:**
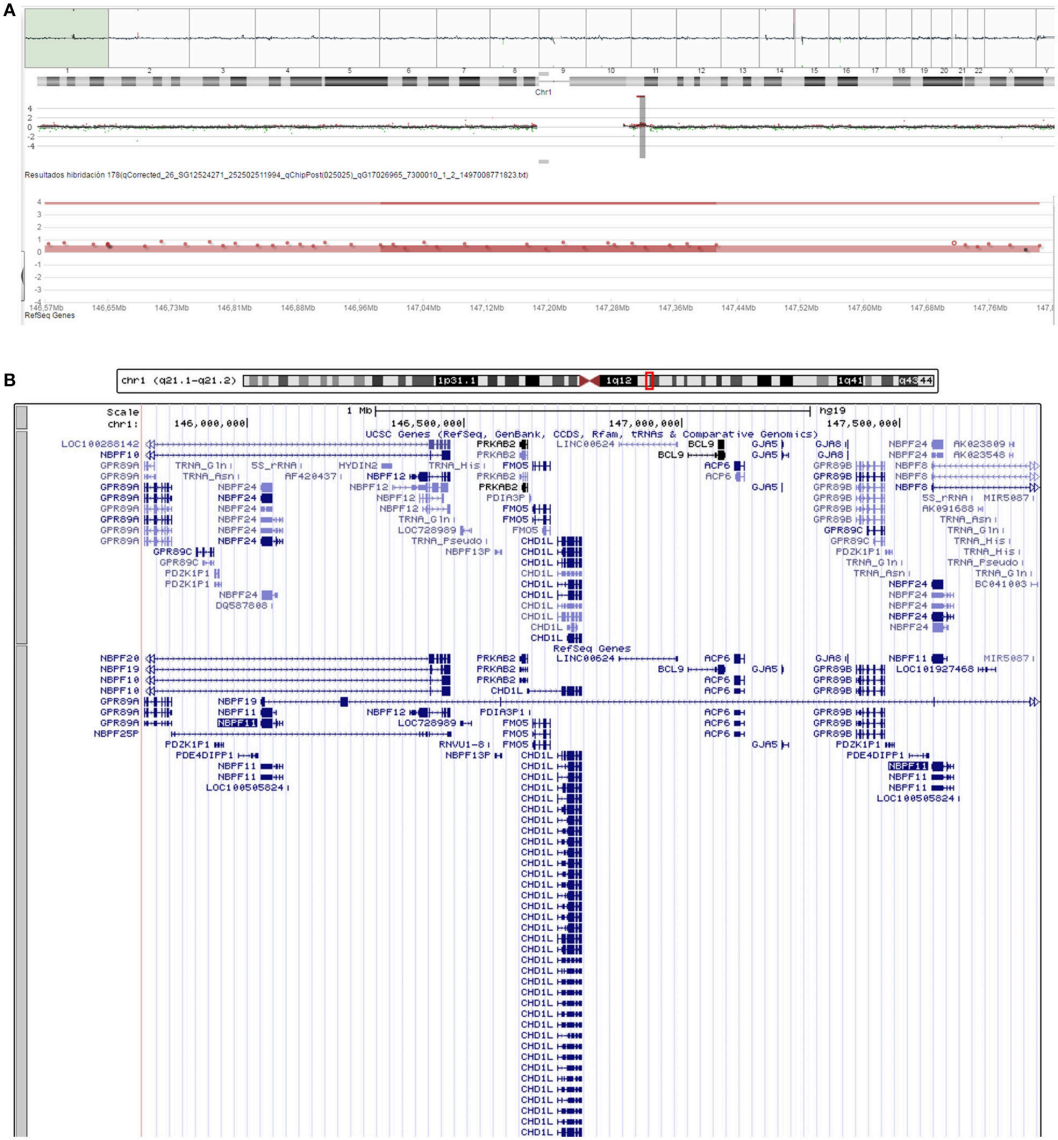
Chromosomal alterations found in our proband. **(A)** Screen capture of the array-CGH of the proband's chromosome 1 showing the microduplication at 1q21.1. **(B)** Screen capture of the UCSC Genome Browser (https://genome.ucsc.edu/) showing the genes duplicated in the proband.

Routine cytogenetic and molecular analyses of the proband were performed when he was 2 years and 7 months old. PCR analysis of the FMR1 fragile site was normal. MLPA of subtelomeric regions was normal too. A comparative genomic hybridization array (array-CGH) identified a duplication of 2.1 Mb in the 1q21.1 region (arr[hg19] 1q21.1-q21.2(145,764,455-147,824,207) × 3) (Figure 2A). The duplicated region corresponds to the region duplicated in Class II 1q21.1 duplication syndrome and includes 38 genes of which 13 are protein-coding genes (Figure 2B).

**Figure 3:**
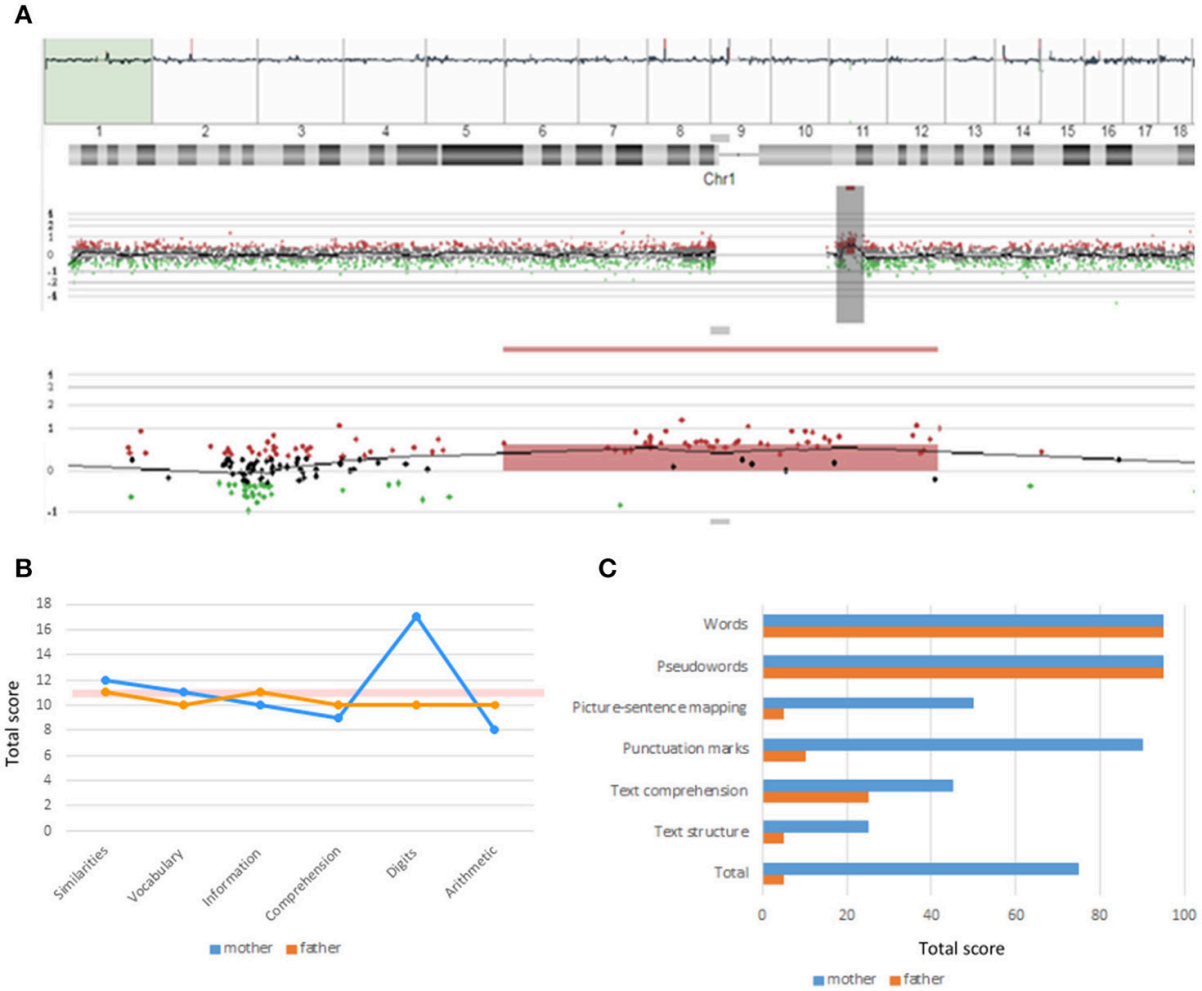
Chromosomal alterations and linguistic profile of the proband's mother. **(A)** Screen capture of the array-CGH of the chromosome 1 of the proband's mother showing the microduplication at 1q21.1. **(B)** Developmental profile of the proband's mother according to the verbal component of the WAIS-III (for comparison, the figure includes the profile of the healthy, non-carrier father). The pink horizontal bar shows the normal values. **(C)** Reading abilities of the proband's mother according to the PROLEC-SE test (for comparison, the figure includes the profile of the healthy, non-carrier father: the low scores obtained by the father are seemingly explained by his low educational level).

As noted, our proband exhibits most of the physical, behavioral, and cognitive features of the 1q21.1 microduplication syndrome (Table 1). The duplication was inherited from the proband's non-symptomatic mother (Figure 3A). The woman exhibited a verbal IQ slightly above the mean, although with peaks and valleys, which are suggestive of relative strengths in the domain of attention and auditory memory (task Digits) and of relative weaknesses in the domain of symbol processing (task Arithmetic) (Figure 3B). Additionally, she exhibited a very good command of verbal abilities as assessed by the BAIRES test, around percentile 90th, and normal-to-high reading abilities (Figure 3C). We have not assessed other features of the microduplication. The fact that she suffered from anxiety during pregnancy might be indicative of some affectedness in other domains [see (4), Table 2], but as far as language and cognition are concerned, she performed normally.

**Table 1:** Summary table with the most relevant clinical features of our proband compared to other patients with the 1q21.1 microduplication syndrome [source: Unique (https://www.rarechromo.org/), (1, 2, 4)].

**Clinical features**	
**COGNITIVE/BEHAVIORAL FEATURES**	
Autism or autistic behaviors	
Anxiety and mood disorders	x
Attention deficit-hyperactivity disorder (ADHD)	
Mild-to-moderate mental retardation	x
Mild-to-moderate developmental delay	x
Learning disabilities	x
Language disorder	x
**NEUROLOGICAL FINDINGS**	
Fine motor impairment	x
Coordination disorder	x
Hypotonia	x
Articulatory problems/stuttering	x
Seizures	
Sleep disturbances	x
Agility abnormalities	x
**PHYSICAL FEATURES**	
Macrocephaly (occasional craniosynostosis)	
(Mild) dysmorphic facial features	
Frontal bossing	
Wider space between the eyebrows and above the nose	
Mild hypertelorism	x
Small nose	x
Prominent epicanthic folds	
Wide, flat nasal bridge	
Low-set ears	x
Stature below the average	
Scoliosis	
**OTHER MEDICAL PROBLEMS**	
Gastric ulcers	
Heart disease	
Minor genital anomalies (hypospadias, testicles undescended at birth)	

In order to delve into the molecular causes of the speech and language problems exhibited by the child, we surveyed the literature looking for other clinical cases in which language deficits have been linked or associated to the mutation or the dysfunction of any of the genes duplicated in our proband. We found that *CHD1L* is a candidate for autism spectrum disorder (ASD) and attention deficit hyperactivity disorder (ADHD) (25, 26), whereas *PRKAB2* and *BCL9* have been associated to schizophrenia (27–30).

**Figure 4:**
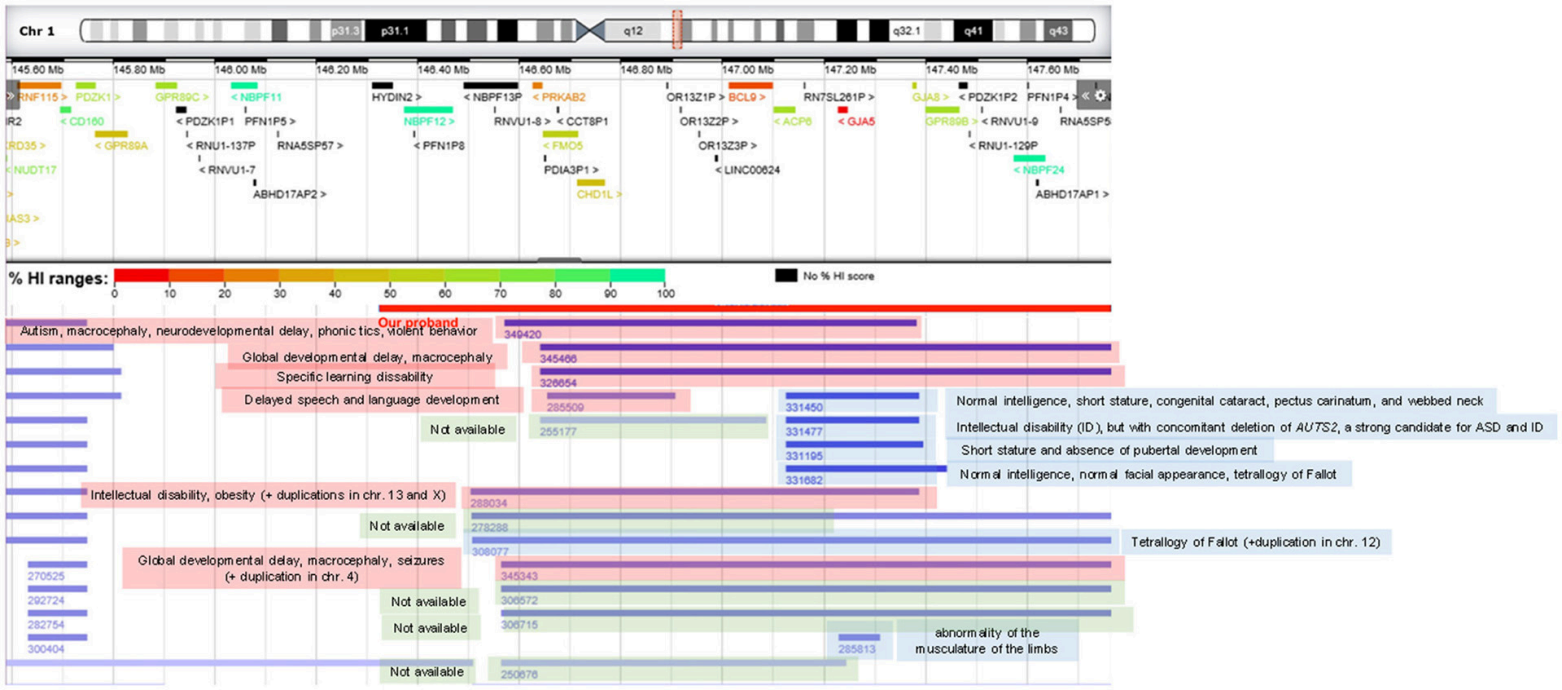
DECIPHER patients of interest bearing chromosomal duplications at 1q21.1 with similar or smaller sizes than the one found in the proband. Patients with cognitive deficits are highlighted in red, cases with no cognitive problems are highlighted in blue, whereas patients for whom no phenotypic profile is available are highlighted in green.

We also relied on DECIPHER (https://decipher.sanger.ac.uk/) to find cases of CNVs affecting chromosomal fragments smaller than the region duplicated in the child, that may help associate language dysfunctions to specific genes. We found that patients bearing microduplications not encompassing the genes *CHD1L* and *BCL9* do not usually exhibit language problems. Conversely, duplications involving *CHD1L* entail delayed speech and language development. No patient bearing a duplication affecting the *BCL9* only has been found (Figure 4).

**Figure 5:**
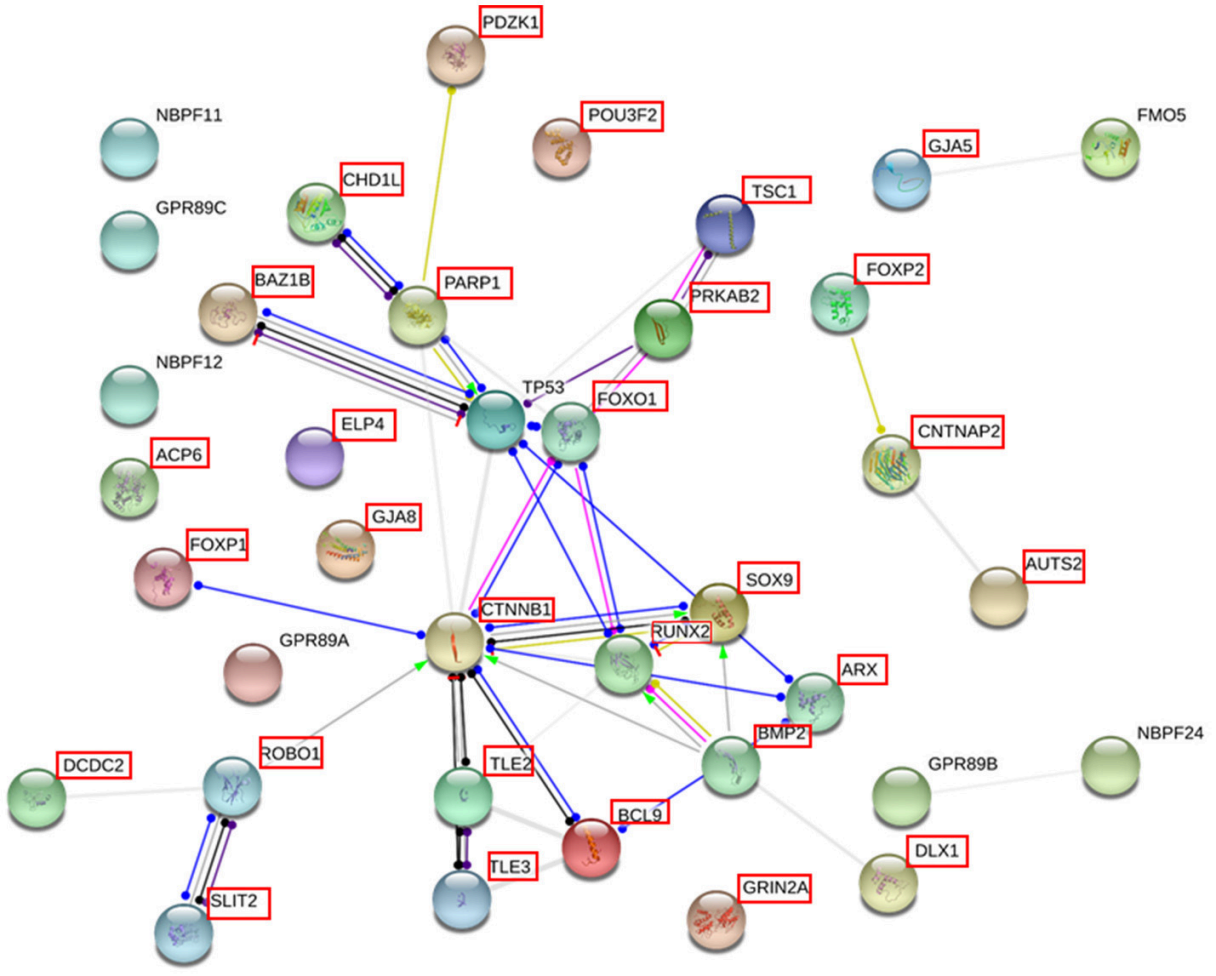
Interaction network of the proteins coded for the most relevant genes duplicated in the proband. The network was drawn with String [version 10.5; (39)] license-free software (http://string-db.org/), using the molecular action visualization. It includes the products of the protein-coding genes duplicated in the subject, their potential interactors according to String, and the products of 15 strong candidates for language development/evolution. The proteins for which the expression level of the gene was measured in the blood of the patient by qRT-PCR are framed in red (the product of the pseudogene *HYDIN2* is not included in the network). Colored nodes symbolize gene/proteins included in the query (small nodes are for proteins with unknown 3D structure, while large nodes are for those with known structures). The color of the edges represent different kind of known protein-protein associations. Green: activation, red: inhibition, dark blue: binding, light blue: phenotype, dark purple: catalysis, light purple: post-translational modification, black: reaction, yellow: transcriptional regulation. Edges ending in an arrow symbolize positive effects, edges ending in a bar symbolize negative effects, whereas edges ending in a circle symbolize unspecified effects. Gray edges symbolize predicted links based on literature search (co-mentioned in PubMed abstracts). Stronger associations between proteins are represented by thicker lines. The medium confidence value was 0.0400 (a 40% probability that a predicted link exists between two enzymes in the same metabolic map in the KEGG database: http://www.genome.jp/kegg/pathway.html). The diagram only represents the potential connectivity between the involved proteins, which has to be mapped onto particular biochemical networks, signaling pathways, cellular properties, aspects of neuronal function, or cell-types of interest.

Finally, we used String 10.5 (www.string-db.org) for examining potential functional links between the genes duplicated in our proband and genes important for language, in particular, known candidate genes for language disorders [dyslexia and SLI, as compiled by (31–33)] and for language evolution, as posited by Boeckx and Benítez-Burraco (34, 35) [importantly, many of these genes are also related to language dysfunction in broader cognitive disorders, particularly, autism and schizophrenia, as discussed in (36–38)] (Supplementary Table 2). String 10.5 predicts physical and functional associations between proteins relying on different sources (genomic context, high-throughput experiments, conserved coexpression, and text mining) (39). Several proteins were predicted to be direct interactors of some of the genes found in the duplicated fragment: FOXO1, TP53, TSC1, for PRKAB2; PARP1, for CHD1L, and CTNNB1, ARX, TL3, and TL2, for BCL9 (Figure 5).

**Figure 6:**
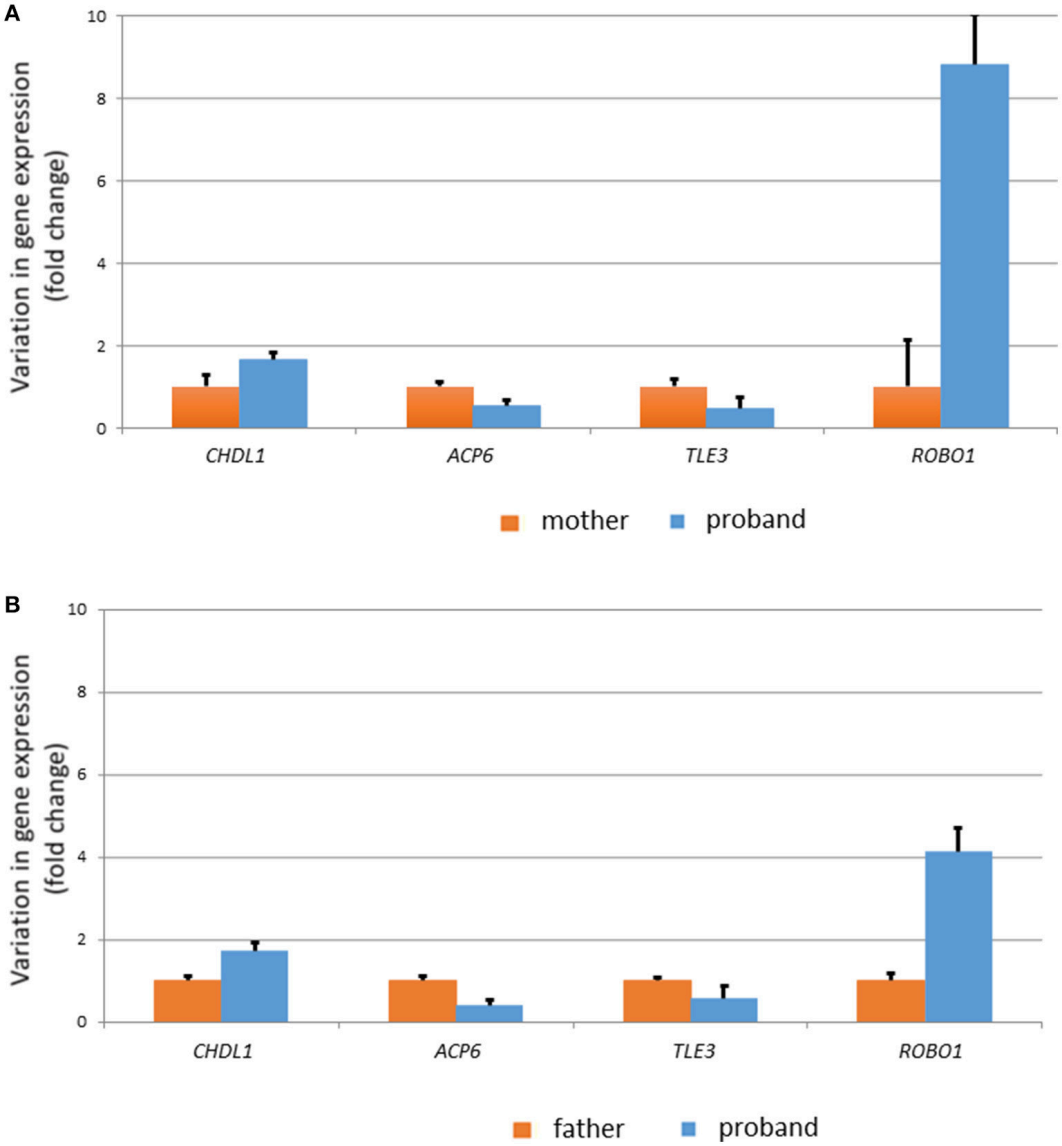
Expression profile of genes of interest in the blood of the proband. Only the genes showing significant (*p* < 0.05) differences with his healthy carrier mother **(A)** and his healthy non-carrier father **(B)** are displayed. Variation in gene expression levels is expressed as fold changes.

In order to check the biological reliability of these potential links and the possibility that other robust candidates for language dysfunction were affected in our proband, we performed qRT-PCR analyses of selected genes using blood from the proband and his parents. We determined the expression level of (i) protein-coding genes located within the duplicated fragment (*BCL9, GJA8, GJA5, ACP6, PDZK1, PRKAB2, CHD1L, HYDIN2*), (ii) functional partners of the protein-coding genes located within the duplicated fragment with a known role in cognition/language development and/or impairment, as found in the literature and as predicted by String 10.5 (*PARP1, CTNNB1, ARX, FOXP2, TLE2, TLE3, FOXO1, TSC1*), and (iii) other strong candidates for language disorders and language evolution, as discussed in Boeckx and Benítez-Burraco (34, 35) (*AUTS2, BAZ1B, BMP2, CNTNAP2, DCDC2, DLX1, ELP4, FOXP1, GRIN2A, POU3F2, ROBO1, RUNX2, SLIT2*, and *SOX9*) (Figure 4). We found that 4 genes are significantly upregulated or downregulated in the proband compared to his healthy parents: *CHD1L, ACP6, TL3*, and *ROBO1* (Figure 6 and Supplementary Table 3).

## Discussion

The widespread application of next generation sequencing facilities to the genetic diagnosis of people with cognitive and language disorders has resulted in a growing number of genes and chromosomal regions associated to these conditions. Nonetheless, in most cases, no robust genotype-to-phenotype correlations have been found. In this paper, we have characterized the cognitive profile of a boy bearing a microduplication in 1q21.1, with a focus on his language deficits. The boy exhibits many of the cognitive, behavioral, and physical symptoms of the 1q21.1 microduplication syndrome (Table 1). Regarding the language (dis)abilities of the affected people, the most detailed account is Bernier et al. (4), who found that ~10% of their subjects suffered from language disorder, whereas phonological processing disorder was observed in ~ 5% of cases. Likewise, around 10% of DECIPHER patients with a duplication within, overlapping, or encompassing the region duplicated in our proband (N = 224) are reported to suffer from language problems. Nonetheless, the linguistic evaluation of patients usually involves psychological tests only. To the best of our knowledge, this is the first time a detailed linguistic profile of a subject with the 1q21.1 microduplication has been provided.

Accordingly, we have conducted a fine-grained analysis of the language strengths and weaknesses of our subject with the 1q21.1 microduplication syndrome using a battery of specific diagnostic tools, as well as examining his language performance in spontaneous conditions, both at the structural and the functional levels. Overall, we found conclusive evidence of a global delay, which plausibly results from delayed and atypical language development in the expressive and receptive domains. The proband's language impairment would be on the basis of a learning disability that precludes the normal acquisition of executive functions and adaptive behavior, hindering the child's interaction with his social environment.

The expressive problems exhibited by the child seem to be also due to altered speech processes, in the form of dysphemia (frequent repetitions or prolongations of sounds and syllables, as well as frequent pauses interrupting the flow of the discourse) and dysprosody (abnormal presentation of prosodic features, like stress, intonation, melody, or rhythmic patterns), as could be observed during the natural spontaneous interaction between the child and his mother. These problems might result in part from deficient executive functions, because we have found no evidence of pure motor speech disorders, like apraxia or dysarthria. Finally, our proband seemingly suffers from a mild pragmatic deficit, because of the also attested delay in the mastering of conversational maxims [see (40, 41) for the neurotypical population].

Regarding the molecular causes of the observed symptoms, Dolcetti et al. (3) point out that UCSC gene expression data are suggestive of the involvement of the genes *BCL9, GJA8, PDZK1*, and *PRKAB2* in the neuropsychiatric phenotype of the 1q21.1 duplication syndrome. O'Bleness et al. (6) have pointed out that the region found duplicated in our proband contains several candidates for human-lineage-specific neurodevelopmental changes, including most of the genes encoding DUF1220 protein domains (*NBPF10, NBPF11, NBPF12, NBPF24*), as well as *PDZK1* and *HYDIN2*. Among the genes located within the duplicated fragment, we have found that 2 genes have changed their expression level in the blood of the affected subject compared to his healthy father and his asymptomatic carrier mother. Hence, *ACP6* is significantly dowregulated, whereas *CHD1L* is significantly upregulated (Figure 6).

**Figure 7:**
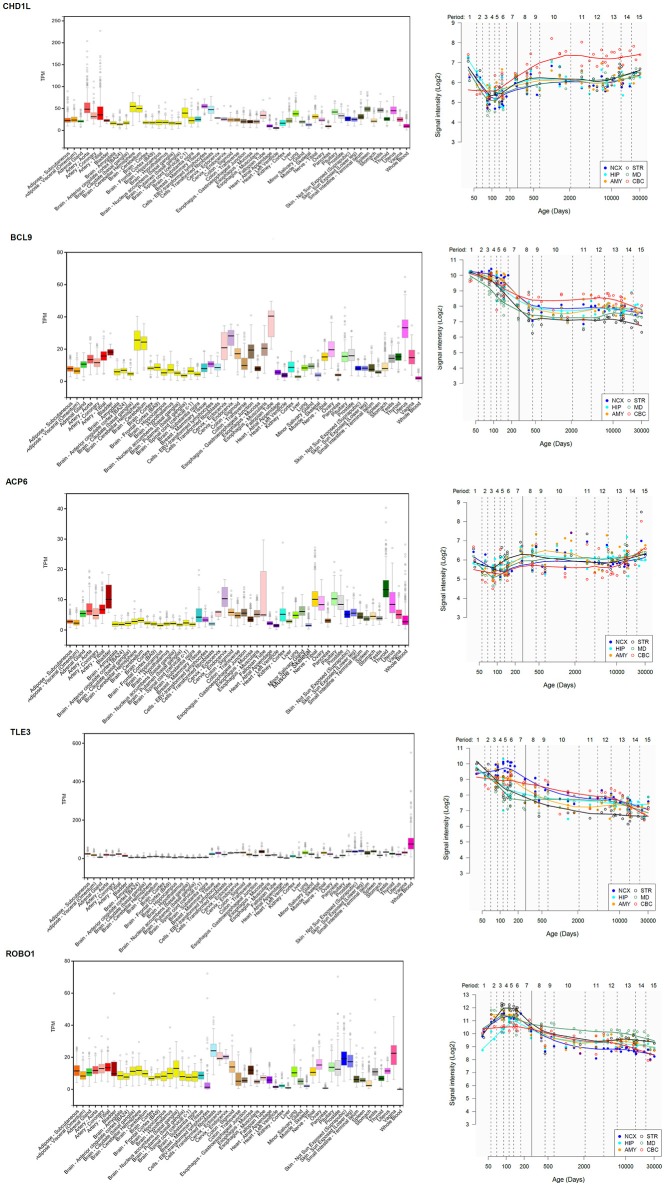
Expression pattern in the body and the brain of genes of interest in the proband. **(Left)** Expression levels of the gene in different tissues according to the Genotype-Tissue Expression (GTEx) project (44). Statistical analysis and data interpretation was performed by The GTEx Consortium Analysis Working Group. Data was provided by the GTEx LDACC at The Broad Institute of MIT and Harvard. TPM, transcripts per million. **(Right)** Brain expression profile of the gene across development. The expression data are from the Human Brain Transcriptome Database (http://hbatlas.org/). Six different brain regions are considered: the cerebellar cortex (CBC), the mediodorsal nucleus of the thalamus (MD), the striatum (STR), the amygdala (AMY), the hippocampus (HIP), and 11 areas of neocortex (NCX).

*ACP6* encodes a histidine acid phosphatase involved in G protein-coupled receptor signaling and the balancing of lipid composition within the cell. Interestingly, differences in the methylation status of the gene have been correlated with differences in hearing abilities associated to age-related hearing impairment (42). Likewise, pathogenic variants in *ACP6* have been recently identified in children with cerebral visual impairment, which results from the impairment in projection and/or interpretation of the visual input in the brain, and which usually co-occurs with intellectual disability (43). The gene is expressed at low levels in the brain (Figure 7).

Regarding *CHD1L*, this gene encodes a DNA helicase protein involved in the relaxation following DNA damage and ultimately, in DNA repair, and its overexpression has been linked to several types of cancers (45). CHD1L is activated by PARP1 during the initial steps of cellular reprogramming (46, 47). Interestingly, *PARP1* is one of the genes that are differentially expressed among (mammalian) vocal learners (48), and its product regulates as well the dyslexia-susceptibility gene *DYX1C1*, important for neuronal migration in the developing cortex (49). We didn't find evidence of a differential expression of *PARP1* in the blood of our patient compared to his mother, who does not exhibit speech or language problems, although the gene is slightly upregulated in both carriers compared to the healthy father (Supplementary Table 3). *CHD1L* is also a candidate for ASD and ADHD (25, 26). Contrary to what is observed in patients with microdeletions of the 1q21.1 region, as the size of duplications encompassing *CHD1L* increases, ASD severity also increases, whereas nonverbal IQ remains unaffected (25). Interestingly, DECIPHER patient 285509, who bears a microduplication affecting only to part of the gene *FMO5* and the whole sequence of *CHD1L*, exhibits delayed speech and language development (Figure 3). *CHD1L* is highly expressed in different brain areas, particularly in the cerebellum, where it is significantly upregulated around birth (Figure 7). The cerebellum is involved in almost every aspect of speech and language processing, from syntax processing to phonological and semantic verbal fluency, verbal working memory and speech perception and planning (50). Moreover, it is involved as well in most of the processes that are impaired in ASD, from language and communication to social interaction and behavior, and cerebellar damage has been found to contribute to the autistic phenotype (51).

We have found in the literature evidence of the potential involvement of *BCL9* in the language deficits observed in patients with the 1q21.1 duplication syndrome. Similarly to *CHD1L, BCL9* is also highly expressed in several parts of the brain, and particularly, in the cerebellum (Figure 7). *BCL9* plays a significant role in embryonic body axis development (52), as part of the Wnt/β-catenin pathway (53). Specifically, BCL9 promotes transcriptional activity and nuclear retention of β-catenin (54). β-catenin is encoded by *CTNNB1*, a gene related to cognitive disorders entailing language deficits, particularly to schizophrenia (55, 56), but also to genes that have been hypothesized to contribute to language evolution in our species [see (34) for details]. *BCL9* itself is a candidate for schizophrenia (27) and interacts as well with genes important for language development and evolution. To begin with, in mice, *Bcl9* is a target of Foxp2, the renowned ‘language gene’ (57). Additionally, BCL9 interacts with ARX to regulate canonical Wnt signaling (58). *ARX* is also a target of FOXP2 (57, 59) and regulates the migration of GABAergic interneurons (60), as well as dopaminergic neuron development (61). In humans, mutations of *ARX* result in mental retardation and interneuronopathies (62), as well as in agenesis of the corpus callosum (63), a condition entailing deficits in problem solving and social skills that often fall within the autistic spectrum (64, 65). Finally, according to the Reactome Pathway Database (https://reactome.org/), BCL9 interacts with both TLE2 and TLE3 as part of the pathway involved in the activation and deactivation of the β-catenin transactivating complex. Both genes encode corepressors of gene expression and play crucial functions in brain development (66, 67). *TLE3* is a target of FOXP2 in the inferior prefrontal cortex (68). Interestingly, we have found that *TL3* is significantly downregulated in the blood of our proband compared to their asymptomatic parents. The gene is upregulated in the embryonic brain, particularly, in the neocortex (Figure 7).

Finally, among the set of robust candidates for language disorders and language evolution, we have found that *ROBO1* is strongly upregulated in the blood of our subject compared to his healthy father and particularly, to his asymptomatic carrier mother. *ROBO1* encodes an axon guidance receptor involved in interneuron migration and axon tract development in the forebrain, in particular, of thalamocortical axons implicated in diverse cognitive functions, consciousness, and alertness (69, 70). Interestingly, *ROBO1* regulates interaural interaction in auditory pathways (71), and was co-opted for vocalization in songbirds as a component of the motor song output nucleus (72). Overall, as part of the SLIT/ROBO pathway, *ROBO1* has been hypothesized to be a crucial candidate for speech development and evolution [see (34) for details]. The gene is a candidate for clinical conditions entailing speech problems, like dyslexia and speech-sound disorder (73, 74). According to its known role in vocalization, the gene is highly expressed in several subcortical regions (Figure 7). No DECIPHER patient bears a duplication of *ROBO1* only, so it is difficult to ascertain the effects in humans of an excessive dosage of the gene. Nonetheless, it is interesting that increased expression of *Robo1* in rats reactivates astrocytes after transient forebrain ischemia (75), whereas the increase of Robo1 function in mice restores normal axon guidance in several neuron populations (76).

In conclusion, although the exact molecular causes of the cognitive and linguistic profile of our proband remain to be fully elucidated, we hypothesize that his distinctive features might result, to a large extent, from the overexpression of *CHD1L*. We further hypothesize that his problems with speech might result from subtle changes in the expression level of some of the genes involved in the externalization of language as part of the FOXP2/ROBO/SLIT regulatory networks, in particular, of *ROBO1*, but also some of the FOXP2's targets, like *TLE2*. We wish to acknowledge two potential caveats of our study. First, we have determined changes in the expression level of genes of interest in the blood, whereas the phenotype under scrutiny has a brain-based locus of impairment. Nonetheless, as shown in Figure 7, our candidates are expressed in the blood and the brain at similar levels. Moreover, several studies point to a significant overlapping in gene expression patterns between both tissues, ranging from 20% (77, 78) to 55% (79). Accordingly, we regard that our findings in the blood can be confidently extrapolated to the brain. Second, because the child was born following a multi-factorial high-risk pregnancy that involved maternal diabetes and exposure to anxiolytic drugs during embryonic and early fetal development, we cannot rule out the possibility that these environmental influences contributed to his language problems. Such an effect is really difficult to estimate. Although recent research has acknowledged diabetes as a robust predictor of language delay in twins (80), the available literature is not fully conclusive [see (81) for a meta-review], and in fact, most children born from mothers with diabetes are not significantly affected if diabetes is properly controlled during pregnancy [see (82) for discussion]. The effect of anxiolytics is perhaps more elusive. Considering the phenotype of our proband, it is interesting that prenatal administration of diazepam delays the maturation of temporal acuity in the rat auditory system (83). This effect might result in altered speech perception in humans. However, no association has been found between anxiolytic exposure during pregnancy and long-term language competence (84). Accordingly, considering as well all the evidence discussed in the paper, we think that the language problems observed in our proband are mostly due to the microduplication in chromosome 1. We hope our findings contribute to a better understanding of the genetic underpinnings of human language.

## Author contributions

AB-B conceived and planned the paper, analyzed the data, and wrote the manuscript. MB-M and IE-P performed the cytogenetic analyses and the microarrays, and analyzed the data. MF-U analyzed in detail the proband's language in use speech and revised the manuscript. RT-R and SR-P performed the qRT-PCRs and analyzed the data. MJ-R assessed the cognitive and linguistic problems of the child, analyzed the data, and revised the manuscript. All authors read and approved the final manuscript.

### Conflict of interest statement

The authors declare that the research was conducted in the absence of any commercial or financial relationships that could be construed as a potential conflict of interest.

## Supplementary material

Supplementary Table 1 List of primers used for qRT-PCR analyses.

Supplementary Table 2 The whole set of genes important for language used for the *in silico* analysis with String 10.5.

Supplementary Table 3 Gene expression levels in the proband and his parents in selected genes of interest.
